# Magnetic field and initial stress on a rotating photothermal semiconductor medium with ramp type heating and internal heat source

**DOI:** 10.1038/s41598-024-64485-8

**Published:** 2024-07-16

**Authors:** Doaa M. Salah, A. M. Abd-Alla, S. M. Abo-Dahab, F. M. Alharbi, M. A. Abdelhafez

**Affiliations:** 1 Department of Mathematics, Faculty of Science, Sohag University, Sohag, Egypt; 2 Department of Mathematics, Faculty of Science, South Valley University, Qena, Egypt; 3 Department of Mathematics, Faculty of Science, Umm Al-Qura University, Makkah, 24227 Saudi Arabia

**Keywords:** Ramp-type heating, Magnetic field, Semiconductor medium, Photothermal, Rotation, Lame’s potential, Initial stress, Normal mode, Solid Earth sciences, Mathematics and computing

## Abstract

This manuscript addresses a significant research gap in the study by employing a mathematical model of photo thermoelastic wave propagation in a rotator semiconductor medium under the effect of a magnetic field and initial stress, as well as ramp-type heating. The considered model is formulated during the photothermal theory and in two-dimensional (2D) electronic-elastic deformation. The governing equations represent the interaction between the primary physical parameters throughout the process of photothermal transfer. Computational simulations are performed to determine the temperature, carrier density, displacement components, normal stress, and shear stress using the application of Lame’s potential and normal mode analysis. Numerical calculations are carried out and graphically displayed for an isotropic semiconductor like silicon (Si) material. Furthermore, comparisons are made with the previous results obtained by the others, as well as in the presence and absence of magnetic field, rotation, and initial stress. The obtained results illustrate that the rotation, initial stress, magnetic field, and ramp-type heating parameter all have significant effects. This investigation provides valuable insights into the synergistic dynamics among a magnetization constituent, semiconducture structures, and wave propagation, enabling advancements in nuclear reactors' construction, operation, electrical circuits, and solar cells.

## Introduction

Researchers have long been interested in the theory of thermoelasticity because of its numerous and significant applications in a variety of disciplines, including the nuclear and aviation industries. In the field of aviation, it has been shown that the high speeds of modern aircraft lead to aerodynamic heating. Consequently, this leads to extreme heat strains and diminishes the airframe's strength by decreasing the elastic limit. Nuclear reactors' construction and operation are impacted by the extremely high temperatures and thermal gradients they produce. Likewise, the high temperatures associated with combustion processes in modern propulsion system technology, such as jet engines and rockets, are the origin of unwanted thermal stresses. Investigation on these and related problems has produced an astounding amount of theoretical and experimental research publications that address different facets of thermal stresses in engineering structures^1–4^. The impact of magnetic fields on solid body deformation, both thermoelastic and elastic, is the subject of the theory of magneto-thermoelasticity.

The Lorentz force, which occurs in the equation of motion, is responsible for the interactions that exist between the electromagnetic field, temperature, stress, and strain in thermoelastic material. This interaction is important because it has many applications in geophysics, such as in plasma physics, the damping of acoustic waves in a magnetic field, electrical power engineering, optics, emission of electromagnetic radiation from nuclear devices, and the influence of the earth's magnetic field on seismic waves. Ezzat^5^ studied the magneto-thermoelastic plane waves with thermal relaxation time in a medium of perfect conductivity. Nayfeh and Nemat-Nasser^6^ discussed how plane waves propagate in a thermoelastic media when they are subjected to an electromagnetic field. Abd-Alla and Abbas^7^ investigated the behavior of stresses, temperature, and magnetic field in an infinitely long, transversely isotropic elastic cylinder using the Lord–Shulman (L–S) and Green–Lindsay (G–L) models. The generalized magneto-thermoelasticity equations in a perfectly conducting material were developed by Ezzat and Youssef^8^ using the Laplace and Fourier transforms approach. The mechanism of photothermal technology is based on two key parts: the first section discusses the principle of thermoelasticity (TE), which happens when a heat wave propagates across a flexible semiconductor material, causing elastic vibrations. Secondly, electronic distortion (ED) is obtained in the flexible semiconductor medium when photoexcited free carriers are produced directly. It has been used to measure several physical parameters of semiconductor materials, including temperature, specific heat, thermal diffusivity, and others^9,10^. Lotfy and Tantawi^11^ investigated the interaction of photothermal elastic waves in a nanocomposite non-homogeneity functionally graded semiconductor elastic material in the presence of an initial magnetic field. Within the context of the theory of two temperatures and the spherical photo-thermal transport process cavity, Mondal and Sur^12^ examined thermal, mixed elastic, and plasma waves within a semiconducting orthotropic infinite media. Applications for problems requiring initially stressed elastic media may be found in a number of fields, including solid mechanics, seismology, geophysics, and earthquake engineering. Many studies have investigated the effects of initial stress on a variety of issues for more details, see^13–15^. Most large bodies such as Earth, moon and other planets have an angle speed, so we need to study the spread of the plane thermoelastic waves in the center of a roundabout with thermal relaxation. Several authors^16–20^ studied the effect of rotation on elastic waves in the circles. Some new contributions in semiconductors with varies external forces was discussed in (Refs.^21–27^).

In this paper, we study the effect of magnetic field and initial stress on the rotating semiconductor medium with ramp-type heating subjected to certain boundary conditions. An external heat source illuminates the surface of a semiconductor material. The basic governing equations is formulated in the $x-y$ plane and in the context of the photothermal theory. Considering the interaction between elastic, plasma, and thermal waves. The physical quantities such as temperature, carrier density, displacement distributions, and thermal stress were obtained using Lame’s potential and normal mode analysis (NMA). Moreover, the MATHIMATICA program will be used to graph and explain the results (in 2D and 3D). Comparisons are made with other papers in the same direction. The results show that magnetic field, rotational velocity, initial stress, and ramp-type heating parameters have significant influence on field variables. The system is extremely valuable for scientists and engineers developing high-quality semiconductor materials that are used in many modern businesses and have various applications in electrical circuits and solar cells (photovoltaics).

## Basic equations and formulation

**Figure 1:**
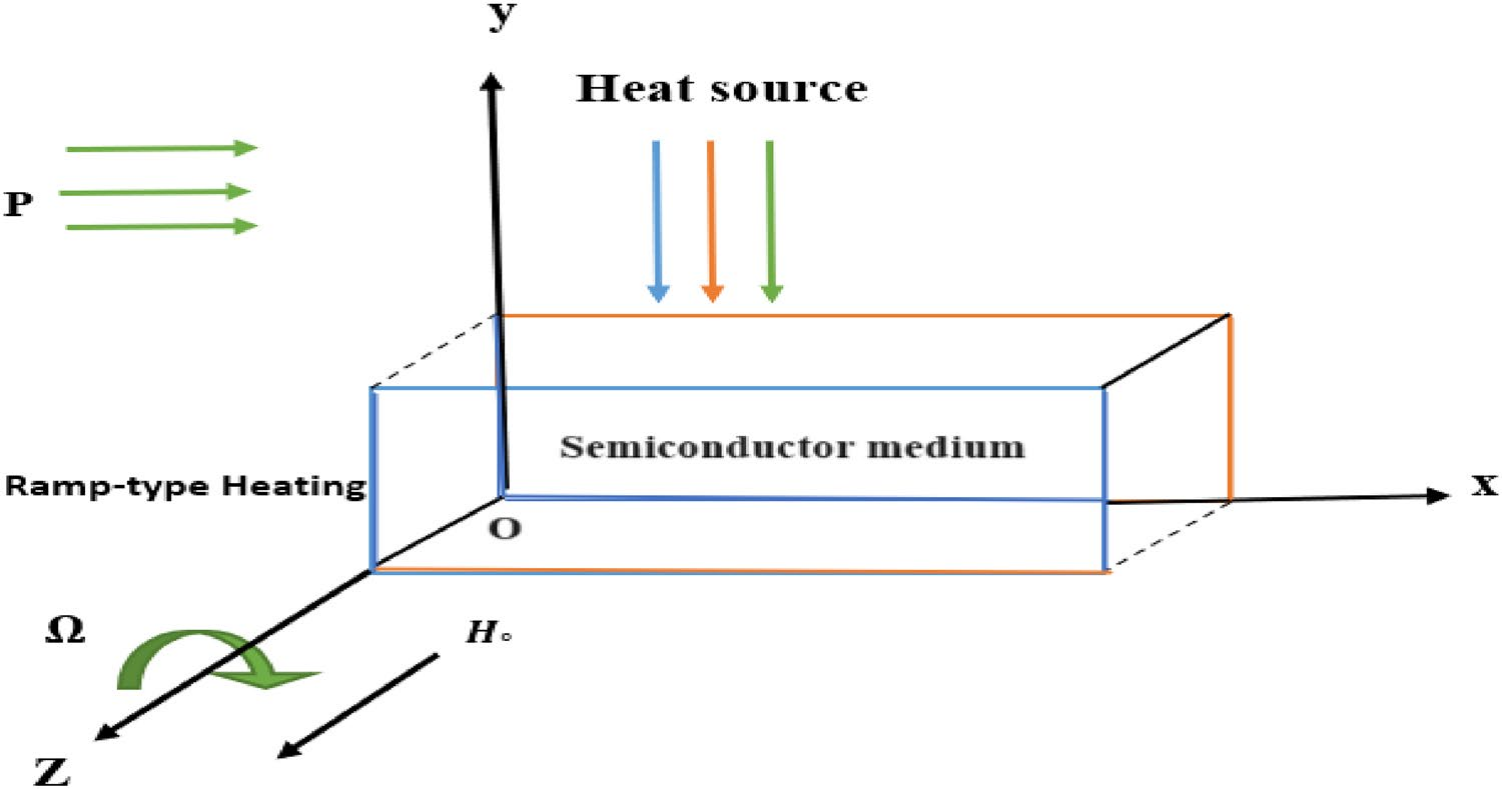
Schematic of the problem.

We consider a homogeneous, isotropic, initially stressed, thermoelastic material with ramp-type heating in the $x-y$ plane (see Fig. 1). Assume that, the medium rotates in a regular manner such that the angular velocity in this case is $\overrightarrow{\Omega }=\Omega \overrightarrow{n}$, where $n$ is a unit vector representing the rotation axis's direction. Consequently, the displacement equation of motion in a rotating frame has two additional terms, Centripetal acceleration $(\overrightarrow{\Omega }\wedge (\overrightarrow{\Omega }\wedge \overrightarrow{u}))$ caused by just time-varying motion, and Cariole's acceleration $(2\overrightarrow{\Omega }\wedge \overrightarrow{\dot{u}})$ caused by a moving reference frame, where $\overrightarrow{u}=(u,v,0)$ is the dynamic displacement vector. Also, the semiconductor medium is exposed to an initial magnetic field $\overrightarrow{\text{H}}=(0,{0,\text{H}}_{0})$ with an induced magnetic field $\overrightarrow{\text{h}}=(\text{0,0},\text{h})$ in the *z*-direction. When the density of charge is ignored an induced electric field $\overrightarrow{\text{E}}$ and a current density vector $\overrightarrow{\text{J}}$ are created in the investigated medium, which fulfill linearized Maxwell's equations.

1 $$\overrightarrow{\text{J}}=\text{curl }\overrightarrow{\text{h}},-{\upmu }_{\text{e}}\frac{\partial \overrightarrow{\text{h}}}{\partial \text{t}}=\text{curl }\overrightarrow{\text{E}},\text{div }\overrightarrow{\text{h}}=0,\text{div }\overrightarrow{\text{E}}=0,\overrightarrow{\text{E}}=-{\upmu }_{\text{e}}\left(\frac{\partial \overrightarrow{\text{u}}}{\partial \text{t}}\times \overrightarrow{\text{H}}\right),\overrightarrow{\text{h}}=\text{curl }\left(\overrightarrow{\text{u}}\times \overrightarrow{\text{H}}\right),$$

From Eq. (1), we can write

2 $$\overrightarrow{\text{E}}=-{\upmu }_{\text{e}}\left(-{\text{H}}_{^\circ }\frac{\partial \text{v}}{\partial \text{t}},{\text{H}}_{^\circ } \frac{\partial \text{u}}{\partial \text{t}},0\right),\overrightarrow{\text{h}}=\left(-{\text{H}}_{^\circ }\text{v},{\text{H}}_{^\circ }\text{u},0\right),\overrightarrow{\text{J}}={-\text{H}}_{^\circ }\left(\frac{\partial \text{u}}{\partial \text{x}}+\frac{\partial \text{v}}{\partial \text{y}}\right).$$

The force of Lorentz $\overrightarrow{\text{F}}$ may be expressed as

3 $$\overrightarrow {{\text{F}}} =\upmu _{{\text{e}}} \left( {\mathop {\overrightarrow {{\text{j}}} }\limits^{{\overrightarrow {{\text{H}}} }} } \right),{\text{ F}}_{{\text{x}}} =\upmu _{{\text{e}}} {\text{H}}_{0}^{2} \left( {\frac{{\partial^{2} {\text{u}}}}{{\partial {\text{x}}^{2} }} + \frac{{\partial^{2} {\text{w}}}}{{\partial {\text{x}}\partial {\text{z}}}}} \right),{\text{F}}_{{\text{y}}} =\upmu _{{\text{e}}} {\text{H}}_{0}^{2} \left( {\frac{{\partial^{2} {\text{u}}}}{{\partial {\text{x}}\partial {\text{y}}}} + \frac{{\partial^{2} {\text{v}}}}{{\partial {\text{y}}^{2} }}} \right).$$

where ${\mu }_{e}$ refers to the magnetic permeability, ${\text{F}}_{\text{x}}$ and ${\text{F}}_{\text{y}}$ are the components of Lorentz force vector $\overrightarrow{\text{F}}$.

For two-dimensional formulation, the constitutive and governing relations that describe the overlapping between elastic-thermal-magneto-plasma waves in semiconductors according to the photothermal theory can be stated as.

The equations of motion without any body forces are:

4 $$\rho \left( {\frac{{\partial^{2} u}}{{\partial t^{2} }} - \Omega^{2} u + 2\Omega \frac{\partial v}{{\partial t}}} \right) = \frac{{\partial \sigma_{xx} }}{\partial x} + \frac{{\partial \sigma_{xy} }}{\partial y} - {\text{P}}\frac{{\partial \omega_{xy} }}{\partial y} +\upmu _{{\text{e}}} {\text{H}}_{0}^{2} \left( {\frac{{\partial^{2} {\text{u}}}}{{\partial {\text{x}}^{2} }} + \frac{{\partial^{2} {\text{v}}}}{{\partial {\text{x}}\partial {\text{y}}}}} \right) - \delta_{n} \frac{\partial N}{{\partial x}},$$

5 $$\rho \left( {\frac{{\partial^{2} v}}{{\partial t^{2} }} - \Omega^{2} v - 2\Omega \frac{\partial u}{{\partial t}}} \right) = \frac{{\partial \sigma_{yy} }}{\partial y} + \frac{{\partial \sigma_{xy} }}{\partial x} - {\text{P}}\frac{{\partial \omega_{xy} }}{\partial x} +\upmu _{{\text{e}}} {\text{H}}_{0}^{2} \left( {\frac{{\partial^{2} {\text{v}}}}{{\partial {\text{y}}^{2} }} + \frac{{\partial^{2} {\text{u}}}}{{\partial {\text{x}}\partial {\text{y}}}}} \right) - \delta_{n} \frac{\partial N}{{\partial x}}.$$

The carrier density equation is:

6 $$\frac{\partial \text{N}(\overrightarrow{r},\text{ t})}{\partial t}={D}_{e}{\nabla }^{2}\text{N}\left(\overrightarrow{r},\text{ t}\right)-\frac{1}{\tau }\text{N}\left(\overrightarrow{r},\text{ t}\right)+\text{k T}\left(\overrightarrow{r},\text{ t}\right).$$

The heat conduction equation is:

7 $$\rho {c}_{e}\frac{\partial \text{T}\left(\overrightarrow{r},\text{ t}\right)}{\partial t}=k{\nabla }^{2}\text{T}\left(\overrightarrow{r},\text{ t}\right)-\frac{{E}_{g}}{\tau }\text{N}\left(\overrightarrow{r},\text{ t}\right)+\upgamma \nabla \cdot \frac{\partial u\left(\overrightarrow{r},\text{ t}\right)}{\partial t}-Q.$$

The stress –displacement relations are:

8 $${\sigma }_{xx}=\left(\lambda +2\mu +P\right)\frac{\partial u}{\partial x}+\left(\lambda +P\right)\frac{\partial v}{\partial y}-\upgamma T,$$

9 $${\sigma }_{yy}=\left(\lambda +2\mu \right)\frac{\partial v}{\partial y}+\lambda \frac{\partial u}{\partial x}-\upgamma T,$$

10 $${\sigma }_{xy}=\mu \left(\frac{\partial u}{\partial y}+\frac{\partial v}{\partial x}\right).$$

From Eqs. (4–7) with the help of Eqs. (8–10) change to

11 $$\rho \left(\frac{{\partial }^{2}u}{\partial {t}^{2}}-{\Omega }^{2}u+2\Omega \frac{\partial v}{\partial t}\right)=\left(\lambda +2\mu +P+{\upmu }_{\text{e}}{\text{\rm H}}_{^\circ }^{2}\right)\frac{{\partial }^{2}u}{\partial {x}^{2}}+\left(\lambda +\mu +\frac{3\text{p}}{2}+{\upmu }_{\text{e}}{\text{\rm H}}_{^\circ }^{2}\right)\frac{{\partial }^{2}v}{\partial x\partial y}+\cdots + \left(\mu -\frac{\text{P}}{2}\right)\frac{{\partial }^{2}u}{\partial {y}^{2}} -\gamma \frac{\partial T}{\partial x}-{\delta }_{n}\frac{\partial N}{\partial x},$$

12 $$\rho \left(\frac{{\partial }^{2}v}{\partial {t}^{2}}-{\Omega }^{2}v-2\Omega \frac{\partial u}{\partial t}\right)=\left(\lambda +2\mu +P+{\upmu }_{\text{e}}{\text{\rm H}}_{^\circ }^{2}\right)\frac{{\partial }^{2}v}{\partial {y}^{2}} +\left(\lambda +\mu +\frac{3\text{p}}{2}+{\upmu }_{\text{e}}{\text{\rm H}}_{^\circ }^{2}\right)\frac{{\partial }^{2}u}{\partial x\partial y}+\cdots +\left(\mu -\frac{\text{P}}{2}\right)\frac{{\partial }^{2}v}{\partial {x}^{2}}-\gamma \frac{\partial T}{\partial y}-{\delta }_{n}\frac{\partial N}{\partial y},$$

13 $$\frac{\partial \text{N}}{\partial t}={D}_{e}{\nabla }^{2}\text{N}-\frac{1}{\tau }\text{ N}+\text{kT},$$

14 $$\rho {c}_{e}\frac{\partial T}{\partial t}=K{\nabla }^{2}\text{T}-\frac{{E}_{g}}{\tau }\text{N}+\upgamma {T}_{0}\frac{\partial }{\partial t}\left(\frac{\partial u}{\partial x}+\frac{\partial v}{\partial y}\right)-Q.$$

where, ${\omega }_{xy}=\left(\frac{\partial u}{\partial y}-\frac{\partial v}{\partial x}\right)$ is infinitesimal rotation, $\gamma =(3\lambda +2\mu ){\alpha }_{T}$ and ${\delta }_{n}=(3\lambda +2\mu ){d}_{n}$.

The governing equations can be expressed in a more convenient form by employing the following non-dimensional variables.

15 $$\left({x}{\prime},{y}{\prime}\right)=\frac{1}{{c}_{T}{t}^{*}}\left(x,y\right),{t}{\prime}=\frac{t}{{t}^{*}},\left({u}{\prime},{v}{\prime}\right)=\frac{1}{{c}_{T}{t}^{*}}\left(u,v\right),{N}{\prime}=\frac{{\delta }_{n}N}{\left(\lambda +2\mu \right)},{\Omega }{\prime}={t}^{*}\Omega ,{c}_{T}^{2}=\frac{(\lambda +2\mu )}{\rho }, {T}{\prime}=\frac{\gamma T}{(\lambda +2\mu )},{\sigma }_{ij}{\prime}=\frac{{\sigma }_{ij}}{\mu },{t}^{*}=\frac{K}{\rho {c}_{e}{c}_{T}^{2}}, {Q}{\prime}=\frac{Q}{\text{Q}0}.$$

Using Eq. (15) in Eqs. (11–14), we have (removing the prime for convenience)

16 $$\left(\frac{{\partial }^{2}u}{\partial {t}^{2}}-{\Omega }^{2}u+2\Omega \frac{\partial v}{\partial t}\right)={a}_{11}\frac{{\partial }^{2}u}{\partial {x}^{2}} +{a}_{12}\frac{{\partial }^{2}u}{\partial {y}^{2}}+{a}_{13}\frac{{\partial }^{2}v}{\partial x\partial y}-{a}_{14}\left(\frac{\partial T}{\partial x}+\frac{\partial N}{\partial x}\right),$$

17 $$\left(\frac{{\partial }^{2}v}{\partial {t}^{2}}-{\Omega }^{2}v-2\Omega \frac{\partial u}{\partial t}\right)={a}_{11}\frac{{\partial }^{2}v}{\partial {y}^{2}} +{a}_{12}\frac{{\partial }^{2}v}{\partial {x}^{2}}+{a}_{13}\frac{{\partial }^{2}u}{\partial x\partial y}-{a}_{14}\left(\frac{\partial T}{\partial y}+\frac{\partial N}{\partial y}\right),$$

18 $${{\frac{\partial \text{N}}{\partial t}=a}_{15}\nabla }^{2}\text{N}-{a}_{16}\text{ N}+{a}_{17}\text{T},$$

19 $${\frac{\partial T}{\partial t}={a}_{18}\nabla }^{2}\text{T}-{a}_{19}\text{ N}+{a}_{10}\frac{\partial }{\partial t}\left(\frac{\partial u}{\partial x}+\frac{\partial v}{\partial y}\right)-{a}_{m}Q,$$

20 $${\sigma }_{xx}={a}_{21}\frac{\partial u}{\partial x}+{a}_{22}\frac{\partial v}{\partial y}-{a}_{23}T,$$

21 $${\sigma }_{yy}={a}_{23}\frac{\partial v}{\partial y}+{a}_{24}\frac{\partial u}{\partial x}-{a}_{23}T,$$

22 $${\sigma }_{xy}=\left(\frac{\partial u}{\partial y}+\frac{\partial v}{\partial x}\right),$$

where

$${\nabla }^{2}=\frac{{\partial }^{2}}{\partial {x}^{2}}+\frac{{\partial }^{2}}{\partial {y}^{2}}$$, $${a}_{11}=\frac{\lambda +2\mu +P+{\upmu }_{\text{e}}{\text{\rm H}}_{^\circ }^{2}}{{c}_{T}^{2}}$$, $${a}_{12}=\frac{\mu -\frac{\text{p}}{2}}{{c}_{T}^{2}}$$, $${a}_{13}=\frac{\lambda +\mu +\frac{3\text{P}}{2}+{\upmu }_{\text{e}}{\text{\rm H}}_{^\circ }^{2}}{{c}_{T}^{2}}$$, $${a}_{14}=\frac{\lambda +2\mu }{{c}_{T}^{2}}$$, $${a}_{22}=\frac{\lambda +P}{\mu }$$, $${a}_{21}=\frac{\lambda +2\mu +P}{\mu }$$, $${a}_{23}=\frac{\lambda +2\mu }{\mu }$$,$${a}_{24}=\frac{\lambda }{\mu }$$, $${a}_{n}=\frac{\text{Q}0\gamma {t}^{*}}{\rho {c}_{e}(\lambda +2\mu )}$$, $${a}_{m}={a}_{n}Q$$.

## Solution of the problem

This section presents the application of Lame's potential and normal mode methods to solve the issue with accuracy and without assuming any constraints on the field variables that are included in the governing equations. Presenting the displacement potentials $\phi (x,y,t)$ and $\psi (x,y,t)$ which are connected to the displacement components by the following relations:

23 $$u=\frac{\partial \phi }{\partial x}-\frac{\partial \psi }{\partial y}, v=\frac{\partial \phi }{\partial y}+\frac{\partial \psi }{\partial x}.$$

The following normal modes can be used to investigate the physical variable solutions:

24 $$\left(u,v,\phi ,\psi ,T,N,{\sigma }_{ij}\right)\left(x,y,t\right)=\left({u}^{*},{v}^{*},{\phi }^{*},{\psi }^{*},{T}^{*},{N}^{*},{{\sigma }_{ij}}^{*}\right)(x){e}^{\left(\omega t+iby\right)}.$$

where the angular frequency, the imaginary number, and the wave number in the y-direction are denoted by the letters $\omega , i, and b$.

Employing Eqs. (23) and (24), Eqs. (16–19) become, respectively:

25 $${(D}^{2}-{m}_{11}){\phi }^{*}-{\gamma }_{12}{T}^{*}-{\gamma }_{12}{N}^{*}=0,$$

26 $${(D}^{2}-{m}_{22}){\psi }^{*}=0,$$

27 $${(D}^{2}-{m}_{33}){N}^{*}+{\gamma }_{19}{T}^{*}=0,$$

28 $${(D}^{2}-{m}_{44}){T}^{*}-{\gamma }_{22}{N}^{*}-{\gamma }_{23}{(D}^{2}+{\text{b}}^{2}){\phi }^{*}-{\gamma }_{24}=0.$$

where $${D}^{2}=\frac{{d}^{2}}{{dx}^{2}}$$, $${\gamma }_{11}=\frac{{a}_{12}+{a}_{13}}{{a}_{11}}$$, $${\gamma }_{12}=\frac{{a}_{14}}{{a}_{11}}$$, $${\gamma }_{13}=\frac{\rho }{{a}_{11}}$$, $${\gamma }_{14}=\frac{\rho {\Omega }^{2}}{{a}_{11}}$$, $${\gamma }_{15}=\frac{{a}_{11}-{a}_{13}}{{a}_{12}}$$, $${\gamma }_{16}=\frac{\rho }{{a}_{12}}$$, $${\gamma }_{17}=\frac{\rho {\Omega }^{2}}{{a}_{12}}$$, $${\gamma }_{18}=\frac{{a}_{16}}{{a}_{15}}$$, $${\gamma }_{19}=\frac{{a}_{17}}{{a}_{15}}$$, $${\gamma }_{20}=\frac{\omega }{{a}_{15}}$$, $${\gamma }_{22}=\frac{{a}_{19}}{{a}_{18}}$$, $${\gamma }_{23}=\frac{{a}_{10}\omega }{{a}_{18}}$$, $${\gamma }_{24}=\frac{{a}_{m}}{{a}_{18}}$$, $${m}_{11}={\text{b}}^{2}{\gamma }_{11}+{\gamma }_{13}{\omega }^{2}-{\gamma }_{14}$$, $${m}_{22}={\text{b}}^{2}{\gamma }_{15}+{\gamma }_{16}{\omega }^{2}-{\gamma }_{17}$$, $${m}_{33}={\text{b}}^{2}+{\gamma }_{18}+{\gamma }_{20}$$, $${m}_{44}={\text{b}}^{2}-\frac{\omega }{{a}_{18}}$$.

Eliminating ${\phi }^{*}(x)$, ${T}^{*}(x)$, and ${N}^{*}(x)$ from Eqs. (25–28) yields the following sixth order equation

29 $${[D}^{6}+{A}_{11}{D}^{4}+{A}_{22}{D}^{2}+{A}_{33}]\{{\phi }^{*}\left(x\right), {T}^{*}\left(x\right), {N}^{*}\left(x\right)\}={G}_{1}$$

The characteristic equation of Eq. (29) is

30 $${\lambda }^{6}+{A}_{11}{\lambda }^{4}+{A}_{22}{\lambda }^{2}+{A}_{33}=0$$

where, ${A}_{11}={m}_{11}+{m}_{33}+{m}_{44}+{\gamma }_{12}{\gamma }_{23}$, ${A}_{22}={m}_{11}{m}_{33}+{{m}_{11}m}_{44}+{m}_{33}{m}_{44}-{\gamma }_{12}{\gamma }_{23}{m}_{33}+{\text{b}}^{2}{\gamma }_{12}{\gamma }_{23}+{\gamma }_{12}{\gamma }_{19}{\gamma }_{23}+{\gamma }_{19}{\gamma }_{24}$, ${A}_{33}={-m}_{11}{m}_{33}{m}_{44}-{m}_{33}{\text{b}}^{2}{\gamma }_{12}{\gamma }_{23}+{\gamma }_{12}{\gamma }_{19}{\gamma }_{23}{\text{b}}^{2}$, ${\lambda }_{i}, i=\text{1,2},\text{3,4},\text{5,6}$ are the all roots for this equation.

The general solutions of Eqs. (25–28), bound as *x* → ∞, are given by:

31 $${N}^{*}=\sum_{i=1}^{3}{A}_{i}{e}^{-{\lambda }_{i}x}+{G}_{2},$$

32 $${T}^{*}=\sum_{i=1}^{3}{\alpha }_{1i}{A}_{i}{e}^{-{\lambda }_{i}x}+{{\beta }_{1}G}_{2},$$

33 $${\phi }^{*}=\sum_{i=1}^{3}{{\alpha }_{2i}A}_{i}{e}^{-{\lambda }_{i}x}+{{\beta }_{2}G}_{2},$$

34 $${\psi }^{*}={A}_{4}{e}^{-\sqrt{{m}_{22}}x}.$$

where $${G}_{1}={m}_{11}{\gamma }_{19}{\gamma }_{24}$$, $${G}_{2}=\frac{{G}_{1}}{{A}_{33}}$$, $${G}_{3}=\frac{{m}_{33}{G}_{2}}{{\gamma }_{19}}$$, $${G}_{4}=\frac{-{\gamma }_{12}({G}_{2}+{G}_{3})}{{m}_{11}}$$, $${\beta }_{1}=\frac{{m}_{33}}{{\gamma }_{19}}$$, $${\beta }_{2}=\frac{-{\gamma }_{12}({\gamma }_{19}+{m}_{33})}{{m}_{11}{\gamma }_{19}}$$, $${\alpha }_{1i}=\frac{{m}_{33}-{{\lambda }_{i}}^{2}}{{\gamma }_{19}}$$, $${\alpha }_{2i}=\frac{{{\gamma }_{12}(m}_{33}-{{\lambda }_{i}}^{2})+{\gamma }_{12}{\gamma }_{19}}{{\gamma }_{19}({{\lambda }_{i}}^{2}-{m}_{11})}$$, $$i=\text{1,2},3$$.

The displacement components may be calculated in the following manner by using Eqs. (23), (24), (33) and (34) as follows:

35 $${u}^{*}=\sum_{i=1}^{3}{{-\alpha }_{2i}{\lambda }_{i}A}_{i}{e}^{-{\lambda }_{i}x}-ib{A}_{4}{e}^{-\sqrt{{m}_{22}}x},$$

36 $${v}^{*}=\sum_{i=1}^{3}{{ib\alpha }_{2i}A}_{i}{e}^{-{\lambda }_{i}x}+ib{{\beta }_{2}G}_{2}-\sqrt{{m}_{22}}{{A}_{4}e}^{-\sqrt{{m}_{22}}x}.$$

By using Eqs. (20–22), and (31)–(36), the stress components may be determined

37 $${{\sigma }_{xx}}^{*}=\sum_{i=1}^{3}{{{a}_{21}\alpha }_{2i}{{\lambda }_{i}}^{2}A}_{i}{e}^{-{\lambda }_{i}x}+ib{a}_{21}{\sqrt{{m}_{22}}A}_{4}{e}^{-\sqrt{{m}_{22}}x}-\sum_{i=1}^{3}{{{b}^{2}{a}_{22}\alpha }_{2i}A}_{i}{e}^{-{\lambda }_{i}x}-{b}^{2}{a}_{22}{{\beta }_{2}G}_{2}-ib{a}_{22}\sqrt{{m}_{22}}{{A}_{4}e}^{-\sqrt{{m}_{22}}x}-\sum_{i=1}^{3}{{a}_{23}\alpha }_{1i}{A}_{i}{e}^{-{\lambda }_{i}x}-{a}_{23}{{\beta }_{1}G}_{2},$$

38 $${{\sigma }_{yy}}^{*}=\sum_{i=1}^{3}{{ib{a}_{23}\alpha }_{2i}A}_{i}{e}^{-{\lambda }_{i}x}+ib{a}_{23}{{\beta }_{2}G}_{2}-{a}_{23}\sqrt{{m}_{22}}{{A}_{4}e}^{-\sqrt{{m}_{22}}x}+\sum_{i=1}^{3}{{{a}_{24}\alpha }_{2i}{{\lambda }_{i}}^{2}A}_{i}{e}^{-{\lambda }_{i}x}+ib{a}_{24}{\sqrt{{m}_{22}}A}_{4}{e}^{-\sqrt{{m}_{22}}x}-\sum_{i=1}^{3}{{a}_{23}\alpha }_{1i}{A}_{i}{e}^{-{\lambda }_{i}x}-{{a}_{23}{\beta }_{1}G}_{2},$$

39 $${{\sigma }_{xy}}^{*}=\sum_{i=1}^{3}{{-2ib\alpha }_{2i}{\lambda }_{i}A}_{i}{e}^{-{\lambda }_{i}x}+{b}^{2}{A}_{4}{e}^{-\sqrt{{m}_{22}}x}+{{{m}_{22}A}_{4}e}^{-\sqrt{{m}_{22}}x}.$$

## Boundary conditions

The following non-dimensional boundary conditions will be considered to avoid unbounded solutions at infinity while ignoring the positive exponential. The unknown parameters ${A}_{i}$, $i=1, 2, \text{3,4}$ will be determined in this part using the suitable conditions for our problem. Also, we assume that the free surface in our suggested model is traction-free.

(I) The plasma condition (carrier density) during the photo-thermal can be presented as
  40 $$\frac{\partial N}{\partial x}=\frac{c}{{D}_{e}}N \text{at} x=0,$$
(II) The normal stress condition (at the free surface) is
  41 $${\sigma }_{xx}=0 \text{at} x=0,$$
(III) The shearing stress is traction-free, then
  42 $${\sigma }_{xy} =0 \text{at} x=0,$$
(IV) The thermal boundary condition according to ramp-type heating at $x$ = 0 is as follows
  43 $$T=\frac{{T}_{1}t}{{t}_{o}}, 0<t\le {t}_{o}.$$

Applying the conditions in Eqs. (40–43) yields four equations for the constants ${A}_{1}$, ${A}_{2}$, ${A}_{3}$ and ${A}_{4}$.

44 $${S}_{1}{A}_{1}+{S}_{2}{A}_{2}+{S}_{3}{A}_{3}={R}_{m},$$

45 $${S}_{4}{A}_{1}+{S}_{5}{A}_{2}+{S}_{6}{A}_{3}+{S}_{7}{A}_{4}={R}_{n},$$

46 $${S}_{8}{A}_{1}+{S}_{9}{A}_{2}+{S}_{10}{A}_{3}+{S}_{11}{A}_{4}=0,$$

47 $${\alpha }_{11}{A}_{1}+{\alpha }_{12}{A}_{2}+{\alpha }_{13}{A}_{3}={R}_{o}.$$

where $${S}_{1}=-{\lambda }_{1}-\frac{c}{{D}_{e}}$$, $${S}_{2}=-{\lambda }_{2}-\frac{c}{{D}_{e}}$$, $${S}_{3}=-{\lambda }_{3}-\frac{c}{{D}_{e}}$$, $${S}_{4}={a}_{21}{\alpha }_{21}{{\lambda }_{1}}^{2}-{b}^{2}{a}_{22}{\alpha }_{21}-{a}_{23}{\alpha }_{11}$$, $${S}_{5}={a}_{21}{\alpha }_{22}{{\lambda }_{2}}^{2}-{b}^{2}{a}_{22}{\alpha }_{22}-{a}_{23}{\alpha }_{12}$$, $${S}_{6}={a}_{21}{\alpha }_{23}{{\lambda }_{3}}^{2}-{b}^{2}{a}_{22}{\alpha }_{23}-{a}_{23}{\alpha }_{13}$$, $${S}_{7}={iba}_{21}\sqrt{{m}_{22}}-{iba}_{22}\sqrt{{m}_{22}}$$, $${{S}_{8}=-2ib\alpha }_{21}{\lambda }_{1}$$, $${{S}_{9}=-2ib\alpha }_{22}{\lambda }_{2}$$, $${{S}_{10}=-2ib\alpha }_{23}{\lambda }_{3}$$, $${S}_{11}={b}^{2}+{m}_{22}$$, $${R}_{m}=(\frac{c}{{D}_{e}}-1){G}_{2}$$, $${R}_{n}={b}^{2}{a}_{22}{{\beta }_{2}G}_{2}+{a}_{23}{{\beta }_{1}G}_{2}$$, $${R}_{o}=-{{\beta }_{1}G}_{2}$$.

To calculate the constants ${A}_{1}$, ${A}_{2}$,${A}_{3}$ and ${A}_{4}$, Cramer's method is applied as there are three non-homogeneous equation in Eqs. (44–47).

48 $${A}_{1}=\frac{\Delta {A}_{1}}{\Delta },{A}_{2}=\frac{\Delta {A}_{2}}{\Delta },{A}_{3}=\frac{\Delta {A}_{3}}{\Delta }, {A}_{4}=\frac{\Delta {A}_{4}}{\Delta },$$

where

49 $$\begin{aligned} \Delta & = S_{11} S_{3} S_{5} \upalpha _{11} - S_{11} S_{2} S_{6} \upalpha _{11} + S_{10} S_{2} S_{7} \upalpha _{11} - S_{3} S_{7} S_{9} \upalpha _{11} - S_{11} S_{3} S_{4} \upalpha _{12} + S_{1} S_{11} S_{6} \upalpha _{12} \\ & \quad - S_{1} S_{10} S_{7} \upalpha _{12} + S_{3} S_{7} S_{8} \upalpha _{12} + S_{11} S_{2} S_{4} \upalpha _{13} - S_{1} S_{11} S_{5} \upalpha _{13} - S_{2} S_{7} S_{8} \upalpha _{13} + S_{1} S_{7} S_{9} \upalpha _{13} , \\ \Delta A_{1} & = R_{o} S_{11} S_{3} S_{5} - R_{o} S_{11} S_{2} S_{6} + R_{o} S_{10} S_{2} S_{7} - R_{o} S_{3} S_{7} S_{9} - R_{n} S_{11} S_{3} \upalpha _{12} + R_{m} S_{11} S_{6} \upalpha _{12} \\ & \quad - R_{m} S_{10} S_{7} \upalpha _{12} + R_{n} S_{11} S_{2} \upalpha _{13} - R_{m} S_{11} S_{5} \upalpha _{13} + R_{m} S_{7} S_{9} \upalpha _{13} , \\ \Delta A_{2} & = - R_{o} S_{11} S_{3} S_{4} + R_{o} S_{1} S_{11} S_{6} - R_{o} S_{1} S_{10} S_{7} + R_{o} S_{3} S_{7} S_{8} + R_{n} S_{11} S_{3} \alpha_{11} - R_{m} S_{11} S_{6} \alpha_{11} \\ & \quad + R_{m} S_{10} S_{7} \upalpha _{11} - R_{n} S_{1} S_{11} \upalpha _{13} + R_{m} S_{11} S_{4} \upalpha _{13} - R_{m} S_{7} S_{3} \upalpha _{13} , \\ \Delta A_{3} & = R_{o} S_{11} S_{2} S_{4} - R_{o} S_{1} S_{11} S_{5} - R_{o} S_{2} S7S_{8} + R_{o} S_{1} S_{7} S_{9} - R_{n} S_{11} S_{2} \upalpha _{11} + R_{m} S_{11} S_{5} \upalpha _{11} \\ & \quad - R_{m} S_{7} S_{9} \upalpha _{11} + R_{n} S_{1} S_{11} \upalpha _{12} - R_{m} S_{11} S_{4} \upalpha _{12} + R_{m} S_{7} S_{8} \upalpha _{12} , \\ \Delta A_{4} & = - R_{o} S_{10} S_{2} S_{4} + R_{o} S_{1} S_{10} S_{5} - R_{o} S_{3} S_{5} S_{8} + R_{o} S_{2} S_{6} S_{8} + R_{o} S_{3} S_{4} S_{9} - R_{o} S_{1} S_{6} S_{9} \\ & \quad + R_{n} S_{10} S_{2} \upalpha _{11} - R_{m} S_{10} S_{5} \upalpha _{11} - R_{n} S_{3} S_{9} \upalpha _{11} + R_{m} S_{6} S_{9} \upalpha _{11} - R_{n} S_{1} S_{10} \upalpha _{12} + R_{m} S_{10} S_{4} \upalpha _{12} \\ & \quad + R_{n} S_{3} S_{8} \upalpha _{12} - R_{m} S_{6} S_{8} \upalpha _{12} - R_{n} S_{2} S_{8} \upalpha _{13} + R_{m} S_{5} S_{8} \upalpha _{13} + R_{n} S_{1} S_{9} \upalpha _{13} - R_{m} S_{4} S_{9} \upalpha _{13} . \\ \end{aligned}$$

The following dimensionless expressions of physical quantities ($u$, $v$, $N$, $T$,${\sigma }_{xx}$,${\sigma }_{yy}$, ${\tau }_{xy}$) can be derived from Eqs. (31–36) and (24)

50 $$u=\left\{\sum_{i=1}^{3}{{-\alpha }_{2i}{\lambda }_{i}A}_{i}{e}^{-{\lambda }_{i}x}-ib{A}_{4}{e}^{-\sqrt{{m}_{22}}x}\right\}{e}^{\left(\omega t+iby\right)},$$

51 $$v=\left\{\sum_{i=1}^{3}{{ib\alpha }_{2i}A}_{i}{e}^{-{\lambda }_{i}x}+ib{{\beta }_{2}G}_{2}-\sqrt{{m}_{22}}{{A}_{4}e}^{-\sqrt{{m}_{22}}x}\right\}{e}^{\left(\omega t+iby\right)},$$

52 $$N=\left\{\sum_{i=1}^{3}{A}_{i}{e}^{-{\lambda }_{i}x}+{G}_{2}\right\}{e}^{\left(\omega t+iby\right)},$$

53 $$T = \left\{ {\sum\limits_{{i = 1}}^{3} {\alpha _{{1i}} } A_{i} e^{{ - \lambda _{i} x}} + \beta _{1} G_{2} } \right\}e^{{\left( {\omega t + iby} \right)}} ,$$

54 $$\begin{aligned} \sigma _{{xx}} & = \left\{ {\sum\limits_{{i = 1}}^{3} {a_{{21}} \alpha _{{2i}} \lambda _{i} ^{2} A_{i} } e^{{ - \lambda _{i} x}} + iba_{{21}} \sqrt {m_{{22}} } A_{4} e^{{ - \sqrt {m_{{22}} } x}} - \sum\limits_{{i = 1}}^{3} {b^{2} a_{{22}} \alpha _{{2i}} A_{i} } e^{{ - \lambda _{i} x}} - b^{2} a_{{22}} \beta _{2} G_{2} } \right. \\ & \quad \left. { - iba_{{22}} \sqrt {m_{{22}} } A_{4} e^{{ - \sqrt {m_{{22}} } x}} - \sum\limits_{{i = 1}}^{3} {a_{{23}} \alpha _{{1i}} } A_{i} e^{{ - \lambda _{i} x}} - a_{{23}} \beta _{1} G_{2} } \right\}e^{{\left( {\omega t + iby} \right)}} , \\ \end{aligned}$$

55 $${\sigma }_{yy}=\left\{\sum_{i=1}^{3}{{ib{a}_{23}\alpha }_{2i}A}_{i}{e}^{-{\lambda }_{i}x}+ib{a}_{23}{{\beta }_{2}G}_{2}-{a}_{23}\sqrt{{m}_{22}}{{A}_{4}e}^{-\sqrt{{m}_{22}}x}+\sum_{i=1}^{3}{{{a}_{24}\alpha }_{2i}{{\lambda }_{i}}^{2}A}_{i}{e}^{-{\lambda }_{i}x}+ ib{a}_{24}{\sqrt{{m}_{22}}A}_{4}{e}^{-\sqrt{{m}_{22}}x}-\sum_{i=1}^{3}{{a}_{23}\alpha }_{1i}{A}_{i}{e}^{-{\lambda }_{i}x}-{{a}_{23}{\beta }_{1}G}_{2}\right\}{e}^{\left(\omega t+iby\right)},$$

56 $${\sigma }_{xy}=\left\{\sum_{i=1}^{3}{{-2ib\alpha }_{2i}{\lambda }_{i}A}_{i}{e}^{-{\lambda }_{i}x}+{b}^{2}{A}_{4}{e}^{-\sqrt{{m}_{22}}x}+{{{m}_{22}A}_{4}e}^{-\sqrt{{m}_{22}}x}\right\}{e}^{\left(\omega t+iby\right)}.$$

## Numerical results and discussion

To demonstrate the behavior of wave propagation of the main physical variables and the findings gained in the preceding section, we chose silicon (Si) as an example of an elastic semiconductor material. The numerical calculations for the distributions of temperature, carrier density, stress, and displacement components were carried out using MATHIMATICA software to represent the outcomes graphically. The physical constants of the silicon element are listed below^28,29^.

*ρ* = 2330 kg/m^3^, *λ* = 3.64 × 10^10^ N/m^2^, *µ* = 5.46 × 10^10^ N/m^2^, $${T}_{^\circ }$$ = 300 K, *K* = 150 W/m k, $${c}_{e}$$ = 695 m^2^/K, $${E}_{g}=1.11$$ eV, $${D}_{e}=2.5\times 1{0}^{-3}{\text{m}}^{2}{\text{s}}^{-1},{\alpha }_{t}=4.14 \times {10}^{-6}{\text{k}}^{-1},c=2{\text{ms}}^{-1},t=0.1\text{s},$$ $${d}_{n}=9\times 1{0}^{-31},$$ $$\tau =5\times 1{0}^{-5}$$ s.

### 2D representation

**Figure 2:**
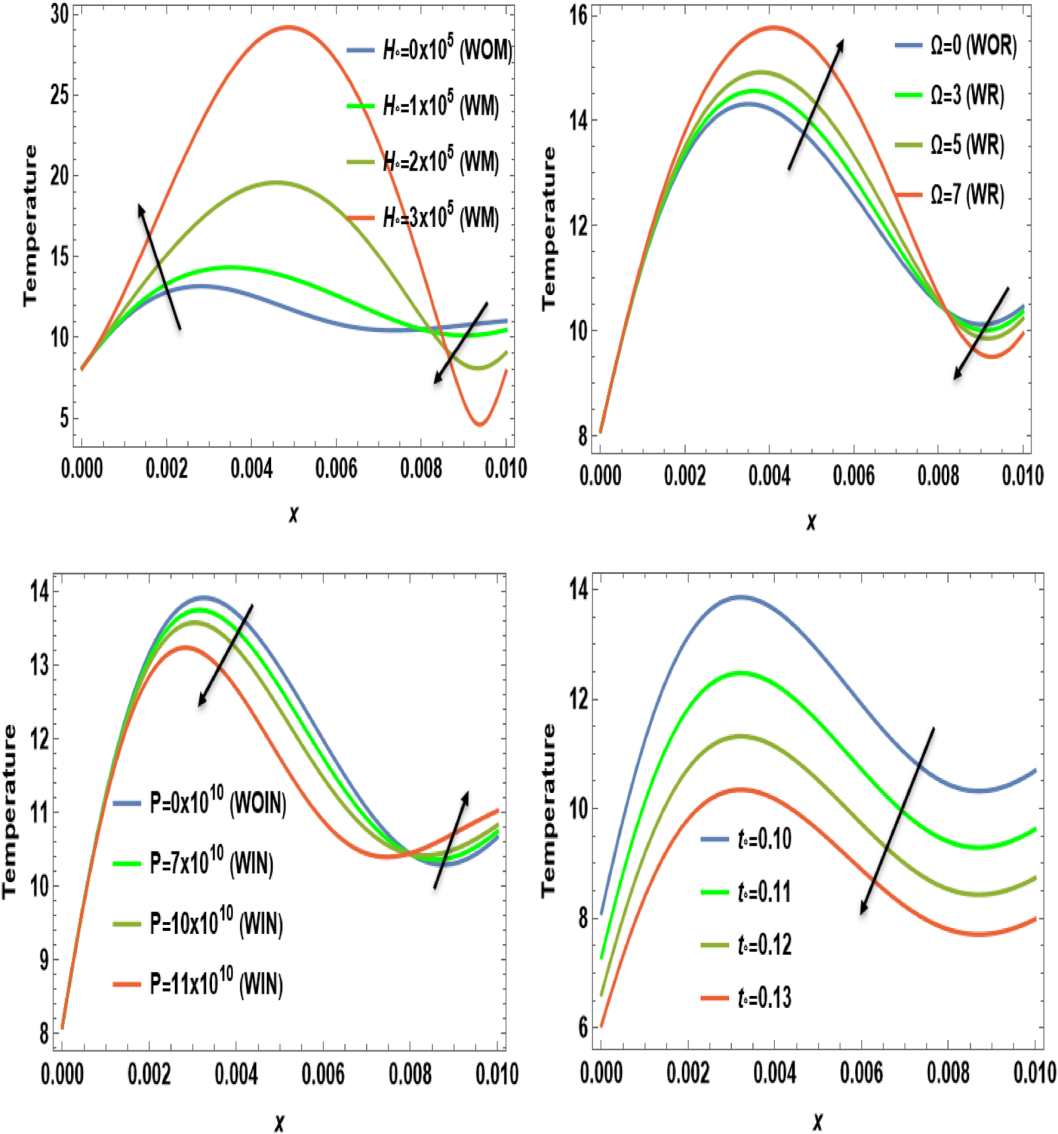
The temperature $T$ distribution versus the distance $x$ during magnetic field, rotation, initial stress, and ramp-type heating parameter effects.

**Figure 3:**
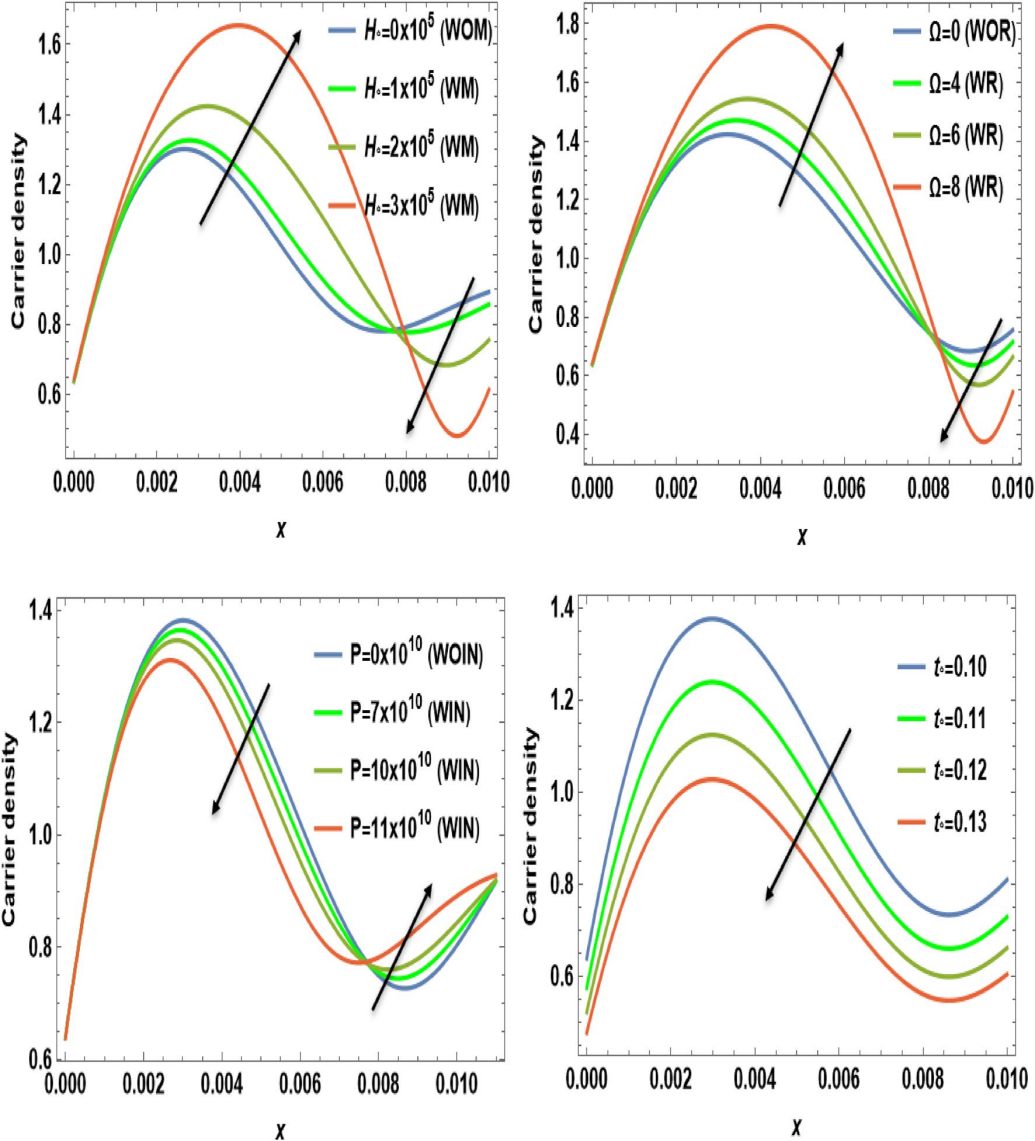
The distribution of carrier density $N$ versus the distance $x$ during magnetic field, rotation, initial stress, and ramp-type heating parameter effects.

**Figure 4:**
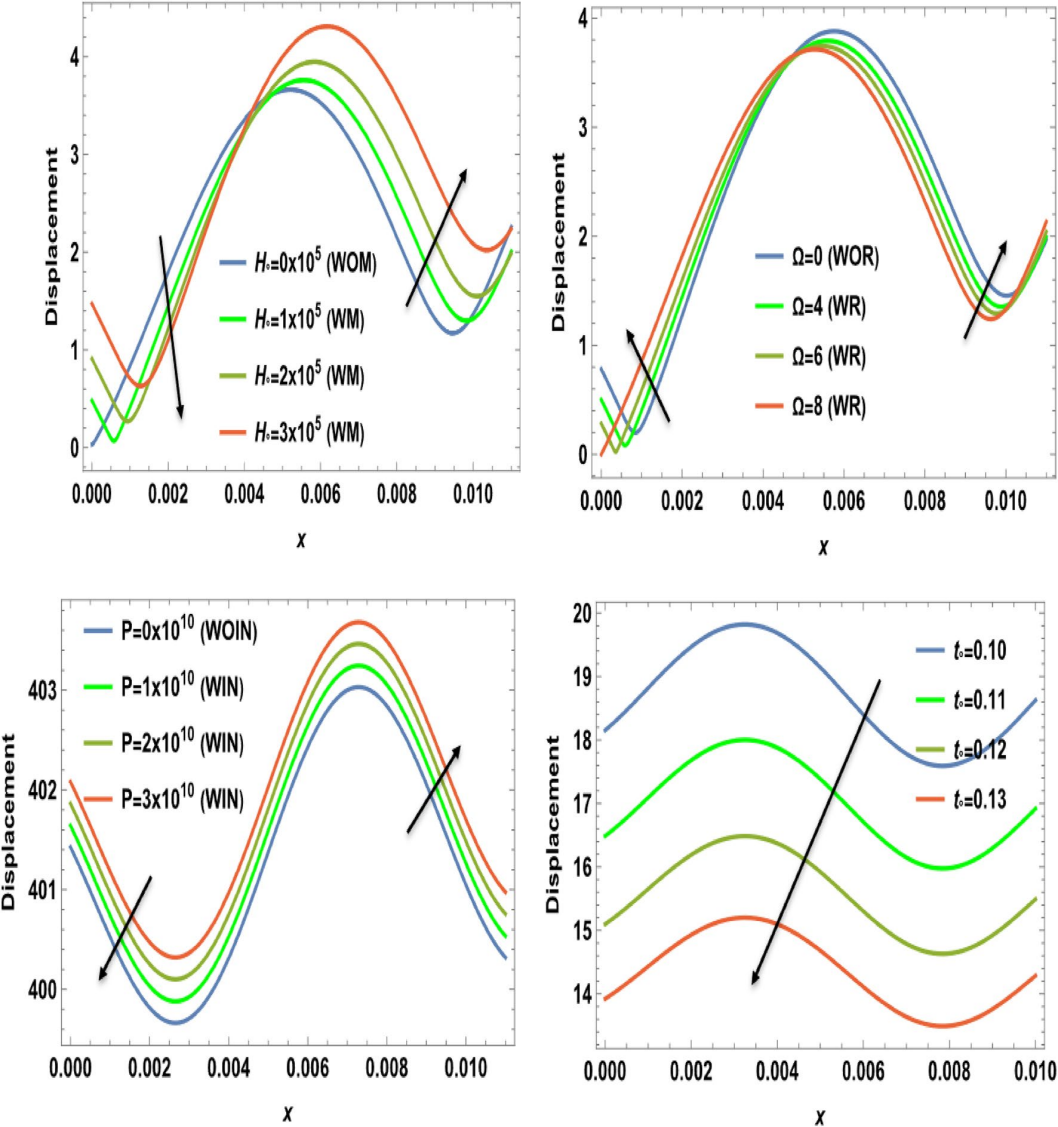
The displacement component $u$ versus the distance $x$ during magnetic field, rotation, initial stress, and ramp-type heating parameter effects.

**Figure 5:**
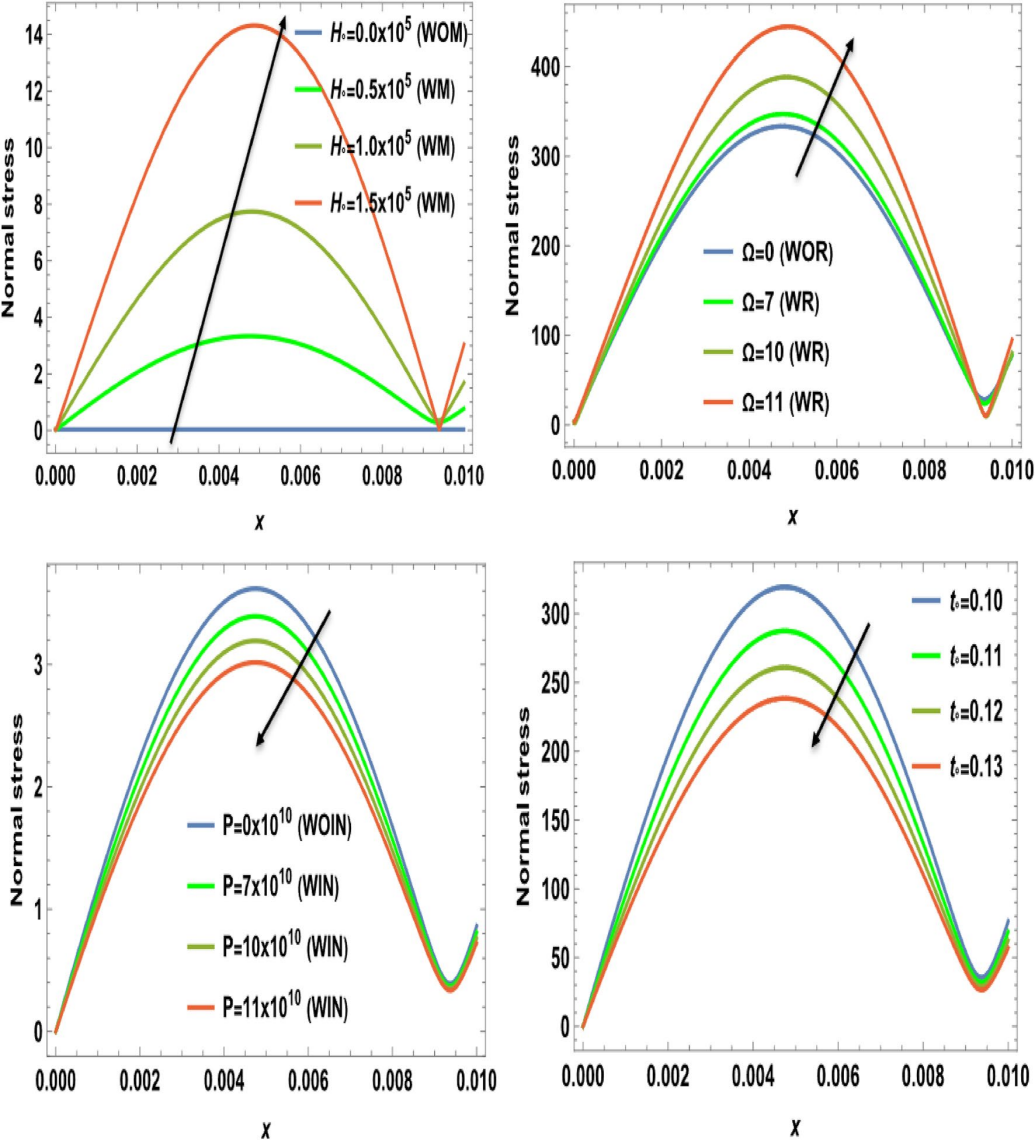
The normal stress component ${\sigma }_{xx}$ versus the distance $x$ during magnetic field, rotation, initial stress, and ramp-type heating parameter effects.

**Figure 6:**
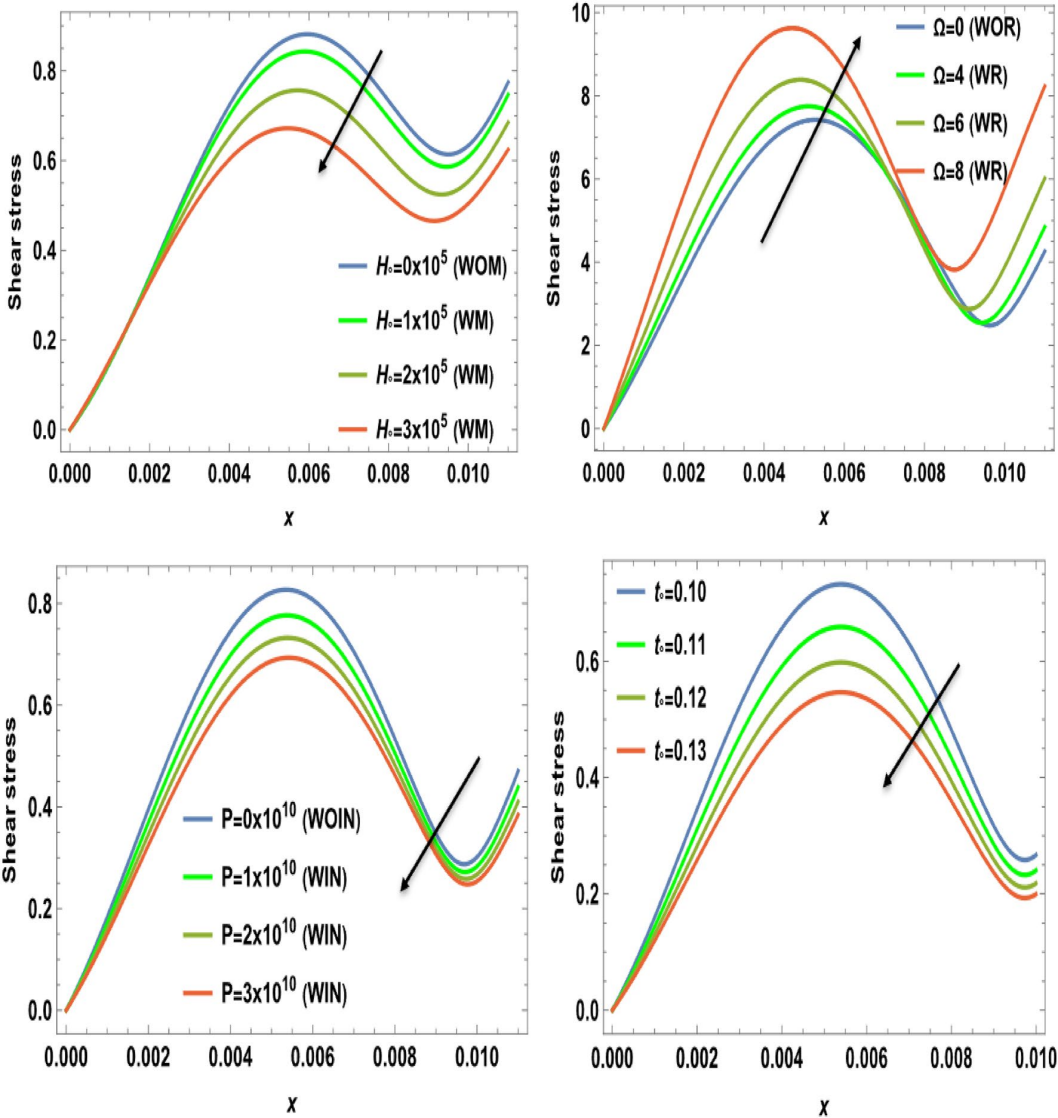
The shear stress component ${\sigma }_{xy}$ versus the distance $x$ during magnetic field, rotation, initial stress, and ramp-type heating parameter effects.

**Figure 7:**
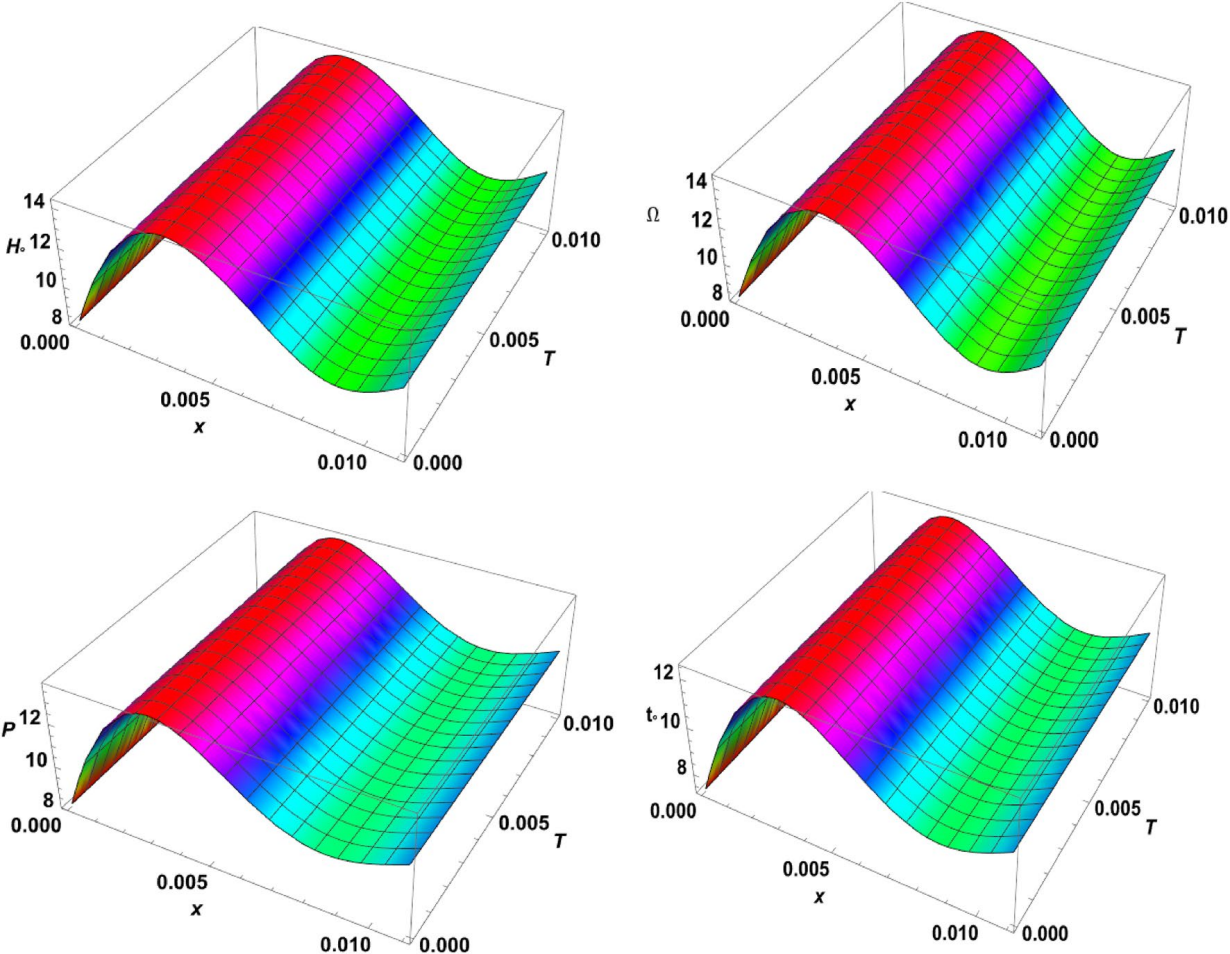
3D-plot for the temperature distribution under the effects of magnetic field, rotation, initial stress, and ramp-type heating against the $x$ and $z$ axes.

**Figure 8:**
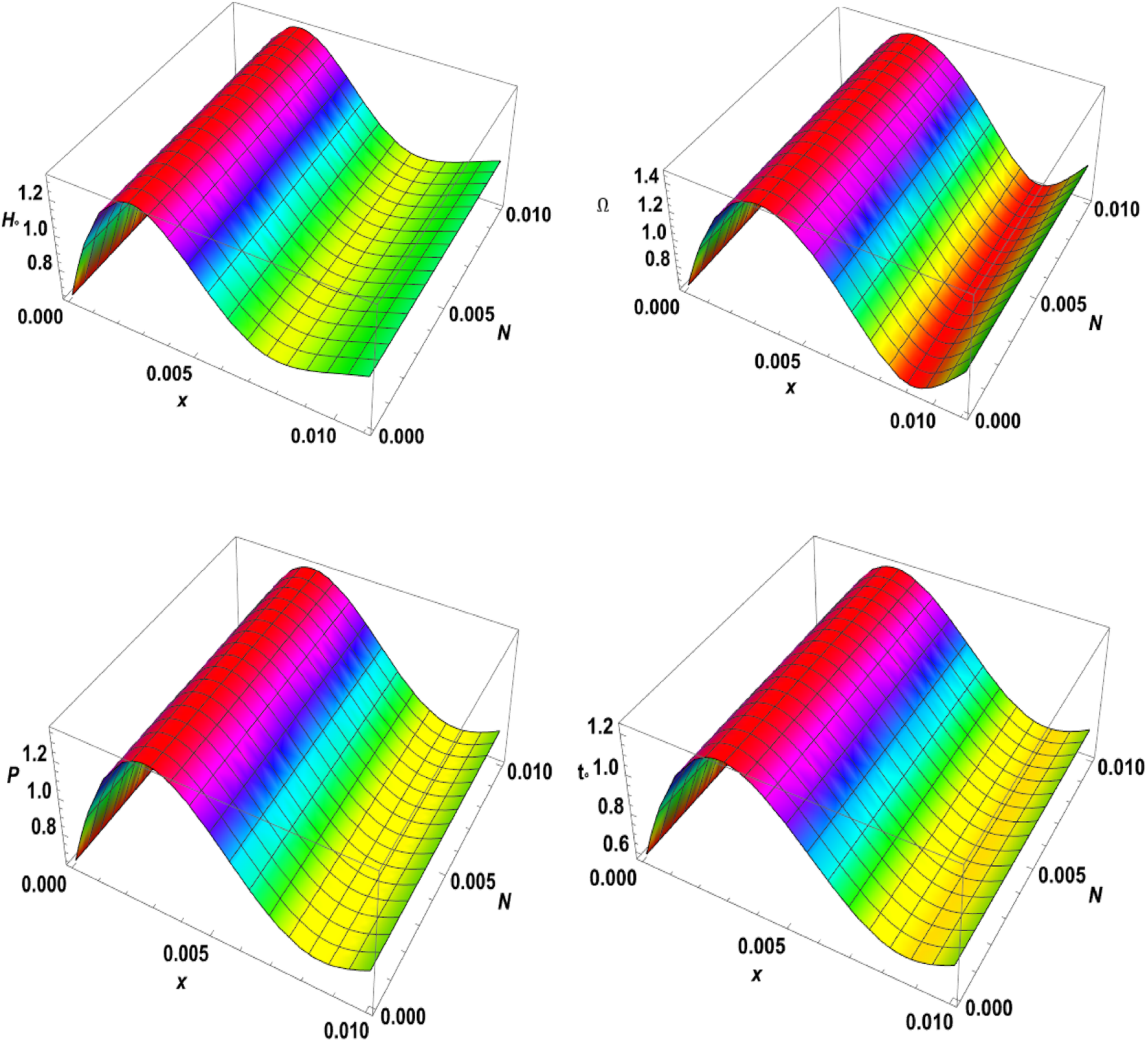
3D-plot for the carrier density distribution under the effects of magnetic field, rotation, initial stress, and ramp-type heating against the $x$ and $z$ axes.

**Figure 9:**
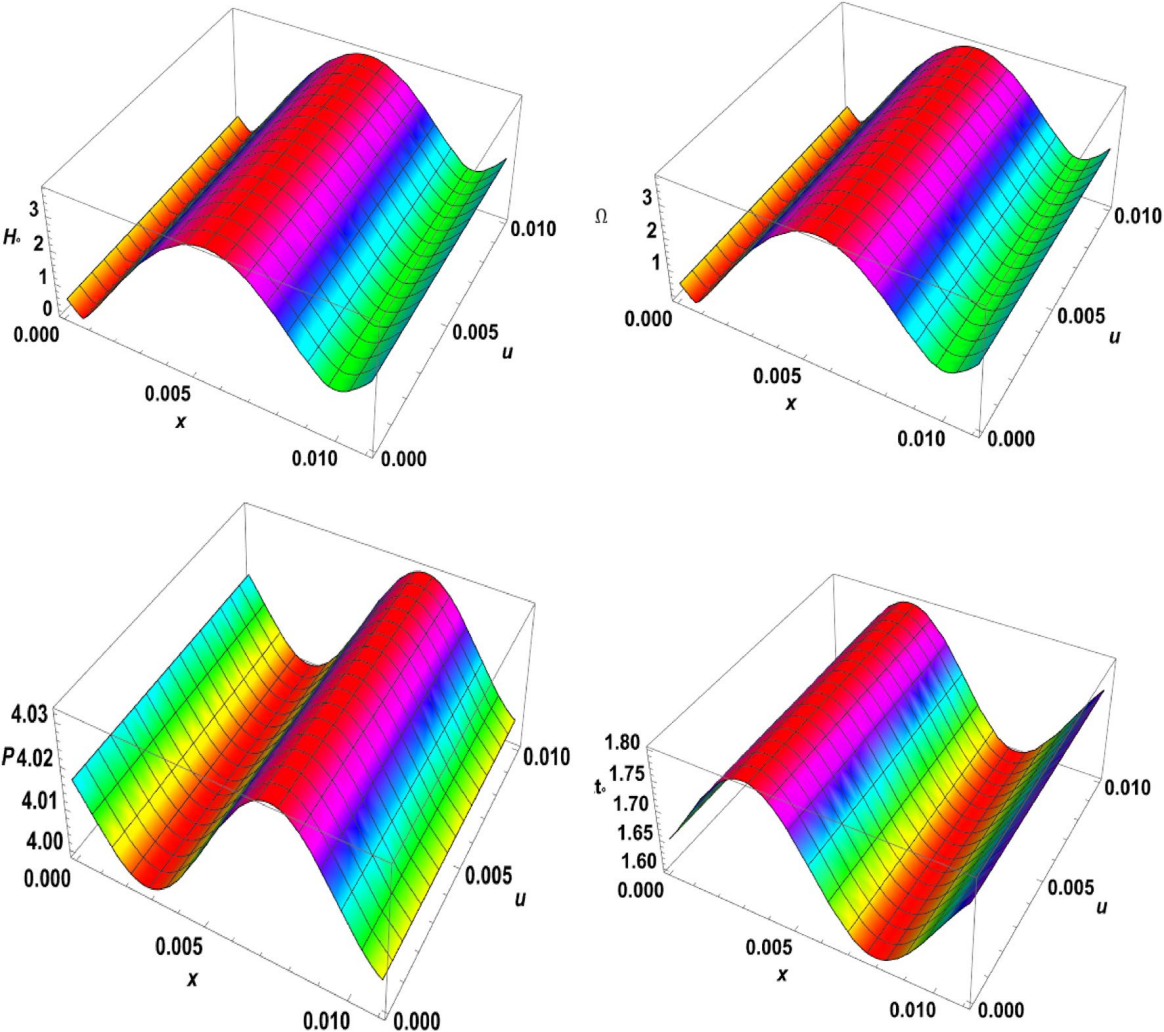
3D-plot for the displacement distribution under the effects of magnetic field, rotation, initial stress, and ramp-type heating against the $x$ and $z$ axes.

**Figure 10:**
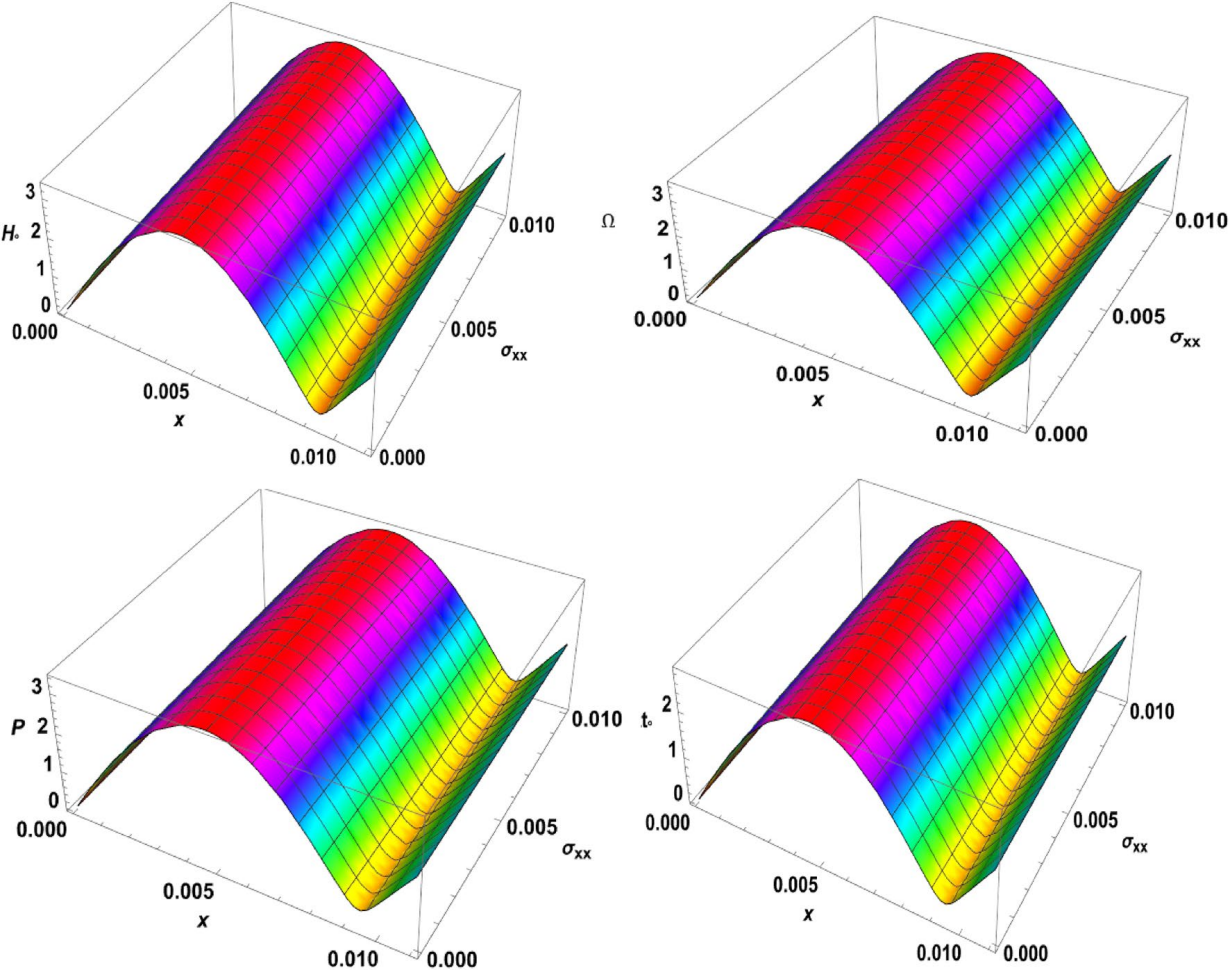
3D-plot for the normal stress distribution under the effects of magnetic field, rotation, initial stress, and ramp-type heating against the $x$ and $z$ axes.

Figures 2, 3, 4, 5, 6, 7, 8, 9 and 10 show numerical and graphical calculations of the temperature, carrier density, thermal stress, and displacement components with distance. Note that the computations have been done at different values in the presence of magnetic field, rotation, and initial stress as well as in the absence of them. Figures 2 and 3 illustrate how the absolute of the temperature and carrier density change with distance based on several factors, including magnetic field, rotation, initial stress, and the ramp-type heating parameter. The magnitude of temperature and carrier density under the effect of both the magnetic field $H_{0}$ and rotation $\Omega$ on the temperature and the carrier density fluctuates. It increases in the period [0, 0.008] and decreases in the period [0.008, 0.01]. The exact opposite happens with the initial stress $P$. It decreases in the period [0, 0.008] and increases in the period from [0.008, 0.01]. But in proportion for the ramp-type heating parameter ${\text{t}}_{\text{o}}$, it decreases over the entire period [0, 0.01]. The research findings indicate that waves exhibit depth-dependent characteristics, which are crucial in understanding their behavior. The study also highlights the correlation between magnetic fields, rotation, and wave propagation, as illustrated in Figs. 2 and 3, while it illustrates the intriguing dynamics of temperature and carrier density change in relation to distance within the examined semiconducture thermoelastic model. Initially, there is a robust ascent in the temperature distribution and carrier density as distance expands, eventually giving way to a gradual descent. This phenomenon demonstrates the ingoing interaction between the propagating wave and the semiconducting medium. The intriguing behavior seen may be due to the intricate characteristics of temperature and other physical quantities, which are well-known for their ability to induce simultaneous harmonic vibrations^31,32^.

Figure 4 displays the effects of magnetic field $H_{0}$, rotation $\Omega$, initial stress $P$, and the ramp type heating parameter ${\text{t}}_{\text{o}}$ on the variation of the absolute value for the displacement component against the axial distance $x$. From this category, it is clear that in the interval [0, 0.004], the amplitude of displacement under the effect of both the magnetic field $H_{0}$ and initial stress $P$ decreases and increases in the interval [0.004, 0.01]. The importance of these discoveries lies in their implications for the behavior of waves in semiconducture magnetization-thermo-elastic mediums. The authors demonstrate that the selection of a parameter value significantly impacts the extent and behavior of particle mobility on the semiconducure medium. As well, it increases in the interval [0, 0.004] and decreases in the interval [0.004, 0.01] under the influence of rotation $\Omega$. But because of the ramp-type heating parameter ${\text{t}}_{\text{o}}$, the displacement distribution values decrease dramatically as horizontal distance rises. This behavior seamlessly aligns with the expected characteristics of displacement components within semiconducture mediums, Hobiny et al*.*^32^, wherein higher harmonic vibrations invariably correlate with more rapid wave propagation. It is observed that the displacement component *u* exhibits oscillatory behavior in the region 0 ≤ x ≤ 0.1 and is greatly influenced by $H_{0}$, $\Omega$, $P$, and ${\text{t}}_{\text{o}}$. Due to the photothermal effect, the semiconductor thermoelastic waves (described by the displacement component u) on the surface are generated with a positive amplitude, which starts reducing when moving away from the source. After that, the semiconductor thermoelastic waves start showing a periodic nature. The effects of magnetic field $H_{0}$, rotation $\Omega$, initial stress $P$, and the ramp type heating parameter ${\text{t}}_{\text{o}}$ on the variation of the absolute value for the normal stress distribution with respect to distance $x$ are shown in Fig. 5. The amplitude of normal stress under the rotational and magnetic field impacts increases in the range $0\le x\le 0.01$. Also, the normal stress decreases by increasing the values of the initial stress and the ramp-type heating parameter in the range $0\le x\le 0.01$. Moreover, as the distance increases, there is a reduction in the amplitude of normal stress. In the starting distance values, the impact of initial stress on normal stress is distinctly prominent. The amplitude of normal stress is seen to have a lower magnitude in the scenario where there is initial stress than in the absence of it. This impact also dims with the increase in distance. In the last figure, Fig. 6 explains the relationship between the change of the absolute value for the shear stress distribution versus the distance $x$ and the magnetic field $H_{0}$, rotation $\Omega$, initial stress $P$, and the ramp type heating parameter ${\text{t}}_{\text{o}}$. The shear stress distributions start from positive values and decrease smoothly nearing the surface in the first horizontal range $0\le x\le 0.01$ until they arrive the minimum value for all four cases due to the effect of magnetic field, initial stress, and ramp-type heating parameter. On the other hand, the numerical calculations begin to increase sharply until they reach maximum values under the rotational effect in the range $0\le x\le 0.01$. After this discussion, we can observe from all the figures that the impact of ramp- type heating parameter on all physical distributions has a great influence on the wave propagations. In addition, the mechanical stresses satisfy the conditions that are applied to the surface. Therefore, their effects must be considered when employing them in technical and practical applications. However, if the magnetic field increases, there is a clear trend of decreasing magnitude in the shear stress. This highlights the impactful relationship between the magnetic field and the resulting amplitude of shear stress. This behavior harmoniously aligns with the anticipated traits of normal stress and shear stress within the semiconductor medium described by Ailawalia^30^. In this context, higher harmonic vibrations consistently correspond to swifter wave propagation.

### 3D representation

Finally, in the 3D representation, Figs. 7, 8, 9, and 10 showcase 3D curves that illustrate the relationship between the physical quantities being studied and both components of distance within the context of the photothermal theory. These quantities are plotted against the horizontal distance $x$ and the vertical distance $z$. The categories explore the impact of the magnetic field ${H}_{^\circ }$, rotation $\Omega$, initial stress $P$, and the ramp type heating parameter ${\text{t}}_{\text{o}}$ on photo-excited processes. It is observed that the magnitude values of the physical fields vary as the $x$ and $z$ axes change. It can be seen that the behaviour of these quantities is the same as the behaviour of wave propagation in 2D. The obtained curves demonstrate a strong dependence on the vertical distance, showing that all the physical quantities are involved in wave propagation. This highlights the dynamic nature of these quantities as they propagate through the medium.

## Particular cases and comparisons

### Neglecting the magnetic field, rotation, and initial stress

Neglecting the magnetic field, rotation, and initial stress effects (i.e., $H_{0}$ = $\Omega$ = $P$ = 0) in Eqs. (11) and (12), the expressions for temperature, carrier density, displacements, and thermal stress distribution reduce to that in the generalized photo thermoelastic medium with internal heat source, leading to findings similar to those found by Praveen^30^ and Abbas^31,32^, as a particular case in this work.

### The presence of magnetic field and rotation

When the magnetic field and rotation are present in the governing equations during the microtemperature process, this implies the results presented by Lotfy^33^, as a special case in this work.

### Neglecting the magnetic field, and initial stress

Neglecting the magnetic field and initial stress effects (i.e., ${H}_{0}=P=0$) in Eqs. (11) and (12), in the specific scenario considered in this study, the formulas for temperature, carrier density, displacements, and thermal stress distribution decrease to those in the generalized photothermal medium with internal heat source, yielding results like this reported by Abd-Alla^34^.

## Conclusion

In the current paper, a two-dimensional problem on wave propagation in a rotating and isotropic thermoelastic medium is presented in the context of the photothermal theory and under the effects of magnetic field and initial stress subjected to ramp-type heating. The governing equations describe the interaction of thermal, plasma, mechanical, and elastic waves during elastic and electronic deformation of a semiconductor material. The formulas for the temperature field, carrier density, displacement components, and stress components were computed numerically using the normal mode technique and displayed graphically (in 2D and 3D). The analytical and graphical results of this study indicate that:

- The model appears more general as it may be used to derive specific examples for additional thermoelastic situations.
- It is noticed from the behavior of wave propagation that, there is a clear influence of magnetic field, rotation, initial stress, and the ramp-type heating parameter on all physical quantities.
- The comparisons were done in two cases. The first case is when the magnetic field, rotation, and initial stress are present in the governing equations and denoted by WMF, WR, and WIN, respectively. The second is when the magnetic field, rotation, and initial stress are absent in the governing equations and denoted by WOMF, WOR, and WOIN, respectively. The impact they have on all physical distributions has a significant effect on wave propagation.
- The magnitudes of the carrier density, displacement, temperature distribution, and stress components are all significantly impacted by the magnetic field. This suggests the physical reality that the phrase denoting positive forces that tend to accelerate metal particles also applies to the influence of a magnetic field.
- The initial stress has an oscillatory behavior on the temperature, carrier density, and displacement variables but decreases with the stress components ${\sigma }_{xx},{\sigma }_{xy}$.
- All of the physical expressions are decreasingly impacted by the ramp-type heating parameter.
- The normal mode technique provides accurate solutions in the generalized thermoelastic medium without assuming any limitations on the real physical quantities that are found in the problem's governing equations.
- The physical variables satisfy all the boundary conditions under investigation.
- This research will help scientists studying thermoelasticity and using rotational motion, which is important in domains such as engineering, physics, robotics, planetary motion, subatomic particle behavior, and machine dynamics.
- Numerical results indicate that, besides mechanical conditions, factors such as magnetic field and rotation significantly affect wave propagation in semiconductor medium. However, it is important to note and take into account all results drawn from a numerical model, which may not accurately reflect the properties of semiconductor materials. Therefore, further experimental verification is necessary before these results can be applied and used in the construction and operation of nuclear reactors, electrical circuits, and solar cells.

## Data Availability

The datasets used and/or analyzed during the current study available from the corresponding author on reasonable request.

## List of symbols

$$\rho$$: Material density
$$t$$: Time
$$T$$: Temperature
$$T_{0}$$: Absolute temperature
$$u,v$$: Displacement components
$\phi ,\psi$: Two scalar functions
${c}_{e}$: Specific heating at constant strain
*N*: Carrier density
$$\lambda , \mu$$: Lame’s constants
${D}_{e}$: Coefficient of carrier diffusions
${\alpha }_{T}$: Coefficient of the linear thermal expansion
$\tau$: Photo-generated carrier lifetime
${d}_{n}$: Coefficient of electronic deformation
${E}_{g}$: Energy gap of the semiconductor parameter
${\delta }_{n}$: Difference of deformation potential of conduction and valence band
k: Thermal conductivity
$${\sigma }_{ij}$$: Stress tensor
$$b$$: Wave number
$$\omega$$: Angular frequency
$${\upomega }_{\text{xy}}$$: Infinitesimal rotation
Q: Internal heat source function
$$\Omega$$: Rotation
$P$: The initial stress
